# Mutual Mate Choice: When it Pays Both Sexes to Avoid Inbreeding

**DOI:** 10.1371/journal.pone.0003365

**Published:** 2008-10-09

**Authors:** Mathieu Lihoreau, Cédric Zimmer, Colette Rivault

**Affiliations:** U.M.R. 6552 Ethos, CNRS-Université de Rennes 1, Campus Beaulieu, Rennes, France; University of Exeter, United Kingdom

## Abstract

Theoretical models of sexual selection predict that both males and females of many species should benefit by selecting their mating partners. However, empirical evidence testing and validating this prediction is scarce. In particular, whereas inbreeding avoidance is expected to induce sexual conflicts, in some cases both partners could benefit by acting in concert and exerting mutual mate choice for non-assortative pairings. We tested this prediction with the gregarious cockroach *Blattella germanica* (L.). We demonstrated that males and females base their mate choice on different criteria and that choice occurs at different steps during the mating sequence. Males assess their relatedness to females through antennal contacts before deciding to court preferentially non-siblings. Conversely, females biased their choice towards the most vigorously courting males that happened to be non-siblings. This study is the first to demonstrate mutual mate choice leading to close inbreeding avoidance. The fact that outbred pairs were more fertile than inbred pairs strongly supports the adaptive value of this mating system, which includes no “best phenotype” as the quality of two mating partners is primarily linked to their relatedness. We discuss the implications of our results in the light of inbreeding conflict models.

## Introduction

A fundamental question raised by the evolution of mating systems addresses the role of each sex in mate choice. This implies understanding the keys used to select mating partners and how and when they are used. For a long time, female mate choice has been the paradigm and the large majority of studies on sexual selection still focus on the role of females because of their higher investment in gamete production and care of offspring [1]–[6]. However, recent evidence suggests that the importance of male mate choice has been underestimated [7]. A growing number of theoretical models predict that members of both sexes should be selective when they incur similar reproductive costs, resulting in assortative pairings of mate quality [8]–[14]. Mutual choosiness is then expected to evolve in species that fulfil at least three conditions: i) the quality of potential mates must vary substantially; ii) reproduction constraints must be low when, for example, encounter rates with potential mates are high; and iii) individuals of both sexes must allocate valuable resources to their reproductive effort so that investing in mating with one partner reduces their ability to invest in other matings. However, empirical data testing and validating these predictions remain relatively scarce [15]–[20].

In many species, individuals base their mate choice on genetic relatedness [21], [22] to optimize genetic compatibility and avoid costly inbreeding and/or outbreeding depressions [23]–[25]. Theoretical models also predict that close inbreeding could be advantageous when the benefits from inclusive fitness exceed inbreeding depression effects [26]–[30]. The advantage for each sex to accept or to avoid inbreeding would thus depend, on the one hand, on the strength of inbreeding depression and, on the other hand, on characteristics of the mating system such as reproductive investment [26], [29]. Theoretical models predict sexual conflict of interest when only one sex receives a net benefit from inbreeding avoidance. Outside this conflictual situation, mutual mate choice should evolve when inbreeding costs are low and/or reproductive investment is asymmetric between sexes thus favouring inbreeding; or when inbreeding costs are high and/or reproductive investment is symmetric between sexes thus favouring outbreeding [30]. Recently, Thünken et al. [31] clearly provided evidence for mutual selectivity by males and females leading to adaptive inbreeding in a cichlid fish with biparental care and no inbreeding depression, suggesting that this species fits the conditions predicted for the first no-conflict situation.

Here, we questioned whether mutual selectivity can lead to adaptive inbreeding avoidance. We estimated independently: i) selectivity of males; ii) selectivity of females; iii) criteria used by each sex to choose their mating partners; iv) when during the mating sequence these criteria were taken into account; and v) reproductive success of pairings. The gregarious cockroach *Blattella germanica* (L.) offers excellent opportunities to investigate these questions. Its mating strategies remain largely unexplored although its mating behaviour and its reproduction physiology have been known for decades [32]. Contrary to *Nauphoeta cinerea* [33], [34], i.e. the only cockroach species for which sexual selection has been consistently studied, neither male nor female *B. germanica* establish dominance hierarchies and intrasexual agonistic interactions do not interfere with their mating success [35]. Our previous investigations revealed that dispersion of adults between aggregates is not the rule [36], [37], suggesting that encounter rates between closely-related potential mating partners are high. Kin recognition by *B. germanica* cockroaches is independent of familiarity and is mediated through antennal contacts with cuticular compounds [38]. While most matings occur between non-siblings [39], the roles of either sex in this mating decision remain unknown.

We argue that *B. germanica* fulfils the three predicted major requirements for the evolution of mutual mate choice as: i) mate quality varies with levels of genetic relatedness between partners; ii) gregariousness facilitates encounters with potential mates and assessment of mate quality; and iii) males invest in long-lasting courtships and costly spermatophores and females invest in costly oothecae. For all these reasons, we hypothesized that both sexes should benefit by avoiding close inbreeding and selecting non-related mating partners. Here, we investigated for the first time, mating preferences of both males and females, by analysing the key steps of the mating sequence in detail. As within natural aggregates cockroaches have the choice between more than one potential mating partner at a time, we evaluated mating preferences in simultaneous mate choice tests that mimic natural situations better than sequential protocols. To take into account behavioural constraints related to each step of the *B. germanica* mating sequence (described below), males and females were tested in different set-ups. Reproductive success of pairings estimated the adaptive value of this mating system.

## Materials and Methods

### *B. germanica* mating sequence

When females reach sexual maturity, they adopt a calling posture and release a volatile sex pheromone that attracts males [40]. Males then establish antennal contacts with the females and, quickly, partners face each other and fence with their antennae [32]. Perception of a non-volatile sex pheromone on the females' cuticle induces males to pursue their courting sequence [41]. Then males turn around in front of the female, raise their wings perpendicularly above their abdomens thus exposing their abdominal tergal glands [42]. Females can be courted simultaneously by several males. Courting males maintain this position until a female licks the tergal gland secretions of one of them and mounts onto his abdomen [43]. The selected male pushes his abdomen further back under the female and grasps her genitalia. Transfer of sperm and formation of spermatophore last for more than 45 min [44]. Spermatophores with high nitrogen contents provide nutritional resources beneficial to females and their offspring [45]. Females mate only once in their lifetime and produce successive oothecae until their death [46]. Each ootheca contained approximately 35 full-siblings nymphs [39].

The succession of behavioural acts during a mating sequence can be interrupted at any moment, when the appropriate stimulus is missing or when the partner does not perform the expected act. We focused on two key steps when each partner has to decide to continue or to stop the sequence: i) antennal contacting that triggers male wing raising and ii) male wing raising that triggers female mounting.

### Experimental animals

All experimental subjects came from our *B. germanica* laboratory stock culture descending from approximately 100 wild individuals collected in Rennes (France) in 1995. Cockroaches were reared in large cages at 25±1°C, under an artificial 12 h light-12 h dark cycle and were provided water, turkey food pellets and cardboard shelters ad libitum. Mature oothecae were collected from freely mated gravid females and placed in individual rearing boxes (80 mm in diameter ×50 mm high) where they hatched. Nymphs remained in groups of siblings until they became adult. Then, they were marked with a spot of paint. From imaginal moult to the beginning of tests, adults from a given ootheca were separated by sex to preserve their virginity but remained grouped to avoid delaying sexual maturation [47]. Experimental individuals were divided into two categories: i) full-siblings from the same ootheca (r = 0.5) called “siblings” and ii) individuals from different oothecae (0≤r<0.5) called “non-siblings”. Familiarity between individuals reared together has no impact on kin discrimination abilities based on cues correlated with genetic relatedness [38]–[39]. Hence, although our experimental design means that related individuals are also reared together we do not expect this common environment to affect subsequent mate preferences.

### Mate choice by males

**Figure 1:**
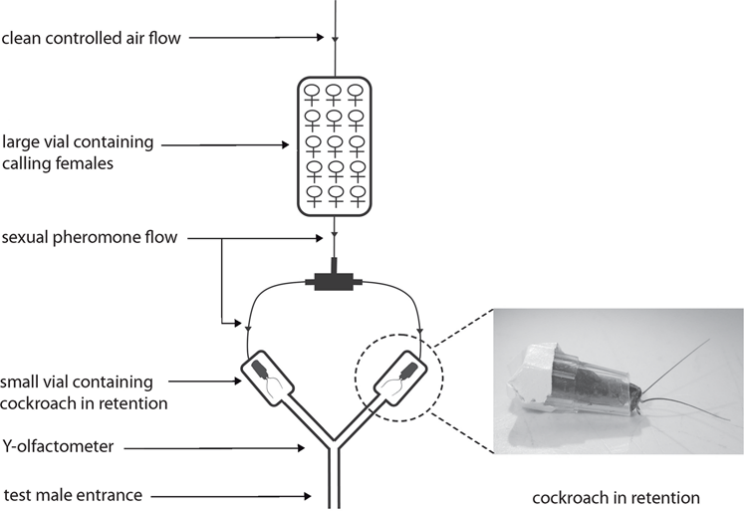
Mate choice by males: glass Y-olfactometer. A male was given a simultaneous choice between two cockroaches in retention, one in each small vial placed at the extremities of the olfactometer. A pump drew clean controlled air through a large vial containing calling females (emitting volatile sex pheromone) and through the olfactometer arms. Cockroaches in retention with free antennae and placed in the flow of sex pheromone constituted calling lures. They could be either males or females.

Each virgin male was given a simultaneous choice between two virgin partners that could be either siblings or non-siblings of the subject. Males were tested in a Y-olfactometer where two calling lures (described below) placed at the end of the arms constituted the two potential mating partners (Figure 1). We used calling lures instead of freely moving calling females to present males a fair choice between two partners differing only by their relatedness, i.e. emitting sex pheromone simultaneously and in similar quantities.

#### Olfactometer design

The glass Y-olfactometer was composed of a starting stem (1 cm internal diameter, 10 cm long) and two arms (1 cm internal diameter, 10 cm long) (Figure 1). A glass stopper connected a cylindrical vial (2 cm internal diameter, 8 cm long) to each arm. Each vial was connected, with Teflon tubes, via a T-connection, to a large glass container (300 ml). At the extremity of the set-up, a pump (New-air, France) pushed charcoal-purified humidified air at a constant flow rate (180 ml/min) controlled by a flow meter (Brooks, USA) through the large glass container and, equally, through the two arms of the olfactometer.

#### Calling lures

Calling lures had to fulfil three conditions: i) both lures had to emit sex pheromone simultaneously to attract males; ii) the sex pheromone had to come equally, i.e. in similar quantities, from two different directions to give males a fair choice between two distinct partners; iii) lures had to be able to perform antennal contacts with test males to induce wing raising. To provide identical sex pheromonal flows through each of the olfactometer arms, 100 potentially calling virgin females were placed in a large glass container to ensure sex pheromone emission during a test (Figure 1). The sex pheromone flow was pushed equally from this large glass container through the two olfactometer arms so that it attracted males to the cylindrical vials at their extremity. To give males a choice between two distinct partners, a cockroach was placed in retention in a small plastic tube (1 cm long, 3 mm in diameter) in each cylindrical vial (Figure 1). One end of the tube was left open so that the head and antennae of the cockroach emerged. Cockroaches in retention were either non-receptive females or males, which meant that none of them emitted female sex pheromone. These cockroaches in retention with free-moving antennae, placed at the end of the olfactometer arms in the sex pheromone flow, constituted calling lures. Lures were either females or males. Relatedness between test males and calling lures was the only independent variable used to test mate choice by males.

#### Behavioural tests

Males were tested in the olfactometer seven days after their imaginal moult. Direct observations recorded the activity of each male for 5 min. A male's choice was estimated by time spent in each arm, time spent antennal contacting each lure and time spent courting (wing raising) in front of each lure. First arm visited and latency of first courting in front of each lure were also recorded.

Four experiments evaluated mate preference by males in relation to their relatedness to the lures. Males were given a choice between: exp. 1a) two sibling female lures (60 replicates); exp. 1b) two non-sibling female lures (60 replicates); exp. 1c) a sibling female lure and a non-sibling female lure (80 replicates); exp. 1d) a sibling male lure and a non-sibling male lure (80 replicates).

### Mate choice by females

Each virgin female was given a simultaneous choice between two virgin males that could be either siblings or non-siblings of the subject. The Y-olfactometer could not be used to test female choice because receptive females adopt a stationary calling posture to attract males. Therefore, each female was tested in experimental boxes (80 mm in diameter ×50 mm high) where the three individuals could move freely and where both males had the possibility to simultaneously court the female. In this set-up, females have the opportunity to exert a choice by actively mounting onto the abdomen of one of the stationary courting males. Although male-male interactions were possible, they were not considered as a potential confounding variable of female choice as there is no evidence that they influence mating success of males [35]. Consequently, the only independant variable used to test mate choice by females was the relatedness between test females and males.

Tests started on the seventh day after female imaginal moult. Scan samples were recorded by direct observation at 30 min intervals, night and day, until mating occurred (range 7–11 days after the imaginal moult). This interval between scans was chosen because mating lasts more than 45 min. Scan data recorded the total numbers of courting attempts by each male until mating occurred and the identity of the male that mated.

Three experiments investigated mate preference of females in relation to their relatedness to males. Females were given a choice between: exp. 2a) two sibling males (45 replicates); exp. 2b) two non-sibling males (170 replicates); exp. 2c) a sibling male and a non-sibling male (79 replicates).

### Reproductive success of pairs

Two hundred and seventy-five females that mated in experiments 2 (exp. 2a: 41; exp. 2b: 160; exp. 2c: 74) were maintained in isolation until their death (range: 61–337 days). To estimate their fecundity, the numbers of viable nymphs hatching from each ootheca were counted. As a female mated with only one male, the total number of nymphs they produced estimated the reproductive success of both mates.

### Statistical analyses

Statistical analyses were performed using R 2.2.1. [48]. Wilcoxon tests compared means of the recorded parameters (time spent in olfactometer arms; antennal contact duration; courting latency; courting duration; number of courting attempts; number of viable nymphs). Binomial tests analysed binary data (arm choice; mate choice). A generalized linear model (GLM procedure; [49]) with binomial errors structure and logit link function analysed the effect of relatedness between males and females on the tendency of males to display courtship (courting attempts). GLMs with Poisson errors and a one-way analysis of variance (ANOVA) investigated the effect of the sex of calling lures on behavioural responses of males (time spent in olfactometer arms; antennal contact duration; courting latency; courting duration). An analysis of covariance (ANCOVA) also evaluated the effects of variables affecting the reproductive success of pairs (female lifespan; relatedness between mates).

## Results

### Mate choice by males

Male mating preference was evaluated by giving males a simultaneous choice in a Y-olfactometer between two calling lures that were either siblings or non-siblings of the test males.

**Figure 2:**
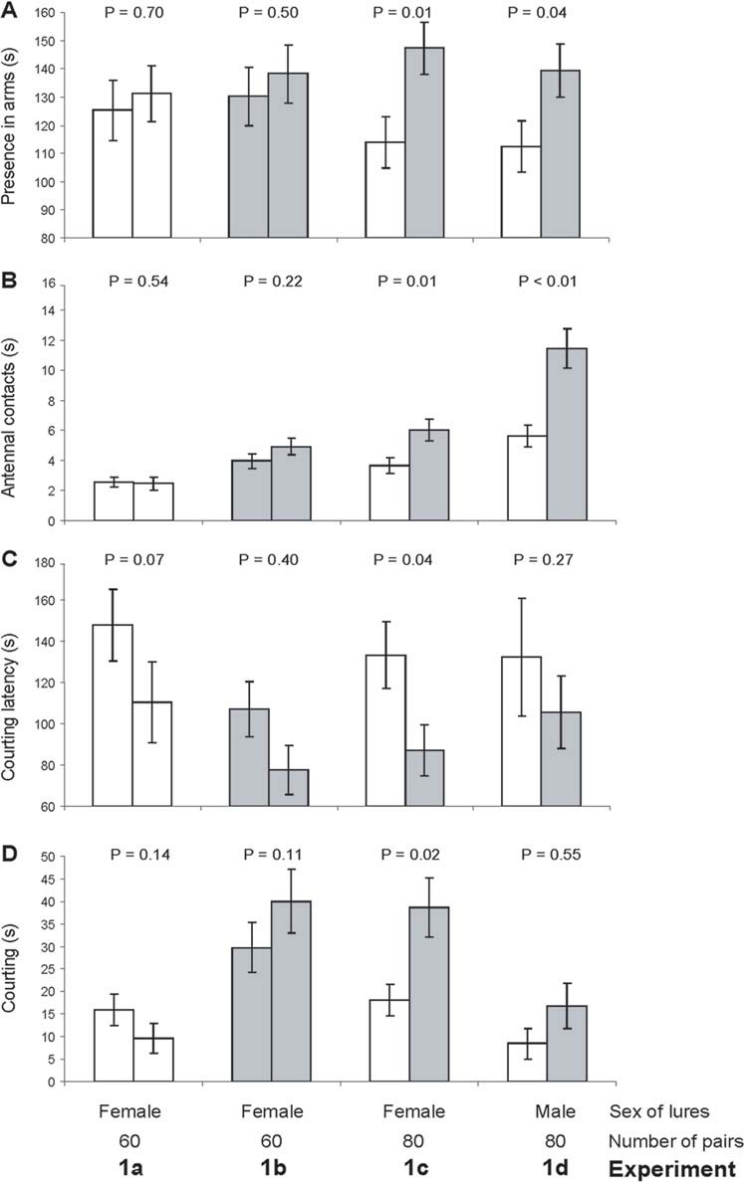
Mate choice by males. Four experiments evaluated mate preferences by males in relation to their relatedness to calling lures. Males were given a choice between: exp. 1a) two sibling female lures; exp. 1b) two non-sibling female lures; exp. 1c) a sibling female lure and a non-sibling female lure; exp. 1d) a sibling male lure and a non-sibling male lure. Test male behaviour was evaluated with: A) time spent with each calling lure in a Y-olfactometer arm; B) antennal contact durations with each calling lure; C) courting latency for each calling lure; D) courting durations in front of each calling lure. Open bars: sibling lures; grey bars: non-sibling lures. Means, in s, ±SE are shown. P = Wilcoxon test.

When given a choice between two sibling female lures (exp. 1a, Figure 2) or two non-sibling female lures (exp. 1b, Figure 2), none of the recorded parameters of the males' courting investment (time spent near a lure, antennal contact durations, courting latencies and courting durations) were biased towards either of the lures. Therefore, when the two female lures did not differ in their relatedness to each other, males courted them equally. Conversely, when given a choice between a sibling female lure and a non-sibling female lure (exp. 1c, Figure 2), males biased all the parameters of their courting investment towards non-sibling female lures. To disentangle the role of males from any influence of females in this decision, test males were given a choice between a sibling male lure and a non-sibling male lure (exp. 1d, Figure 2). In this situation, they spent significantly more time in the olfactometer arm and antennal contacting with the non-sibling male lure than with the sibling male lure (courting latencies and courting durations did not differ significantly). Thus, in the presence of two male lures that differed in their relatedness to each other, test males persistently biased their courting investment toward the non-sibling. Data from these four experiments (exp. 1a–d) revealed that males modified their courting investment in relation to their relatedness to the calling lures, be they females or males. Males always showed a strong preference for non-sibling lures that always induced more vigorous courting displays. As patterns of first visits did not differ significantly between olfactometer arms, even when the relatedness between the two lures differed (binomial test; exp. 1 a: P = 0.25, exp. 1b: P = 0.52, exp. 1c: P = 0.58, exp. 1d: P = 0.43), males did not assess their relatedness to the lure from a distance in the starting stem via an airborne chemical message. This result consequently supports the implication of antennal contacts in kin recognition and suggests that they are a key step in the male's decision to pursue courtship.

Although males biased their courting effort toward non-sibling lures, be they females or males, comparisons between exp. 1c and exp. 1d data revealed that sex of lures influenced the behavioural responses of test males. As expected under our experimental conditions, the calling pheromone flow attracted males from a distance to the end of the olfactometer arms whatever the sex of the lures. Although time spent in an olfactometer arm was not influenced by the sex of calling lures (one-way ANOVA, F_1, 318_ = 0.24, P = 0.63), test males spent longer antennal contacting male lures than female lures (GLM, χ^2^ = 164.8, z = 12.59, P<0.01), courting latency was longer when lures were males than when they were females (GLM, χ^2^ = 12.9, z = 3.6, P<0.01), and males spent less time courting male lures than female lures (GLM, χ^2^ = 1398.4, z = −35.9, P<0.01) (Figure 2). These data indicate that, in addition to relatedness, test males use antennal contact to assess the sex of the encountered lure.

### Mate choice by females

Female mating preference was evaluated by giving them a simultaneous choice between two freely moving males in an experimental box, until mating occurred. Males were either siblings or non-siblings of test females.

**Figure 3:**
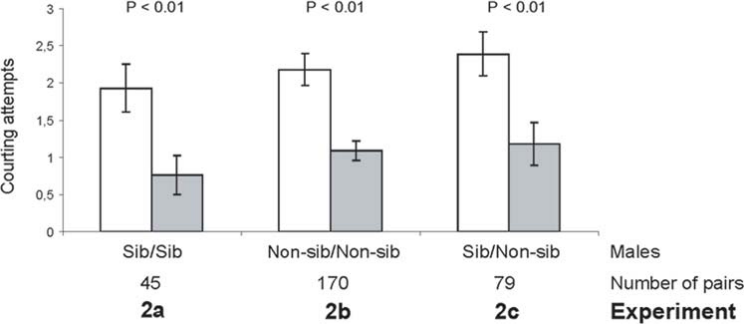
Mate choice by females. Three experiments investigated mate preference of females in relation to their relatedness to males. Females were given a choice between: exp. 2a) two siblings; exp. 2b) two non-siblings males; exp. 2c) a sibling and a non-sibling. Female mate choice was evaluated in relation to the number of courting attempts by males. Open bars: mated males; grey bars: non-mated males. Means±SE are shown. P = Wilcoxon test.

When given a choice between two sibling males (exp. 2a, Figure 3) or two non-sibling males (exp. 2b), females mated with the male that displayed the most courting attempts. Similarly, in the presence of a sibling and a non-sibling male (exp. 2c, Figure 3), females persistently mated with the male that courted them the most vigorously. As non-sibling males performed more courting attempts than sibling males (non-siblings: 2.00±0.28, siblings: 1.16±0.18, W = 2580, P = 0.04), females mated more often with non-siblings (70.89% mating) than with siblings (siblings: 23, non-siblings: 56, binomial test, P<0.01). These data support the fact that females are selective and that they bias their choice towards males performing the most vigorous courtships, these males more often happened to be their non-siblings.

### Reproductive success of pairs

**Figure 4:**
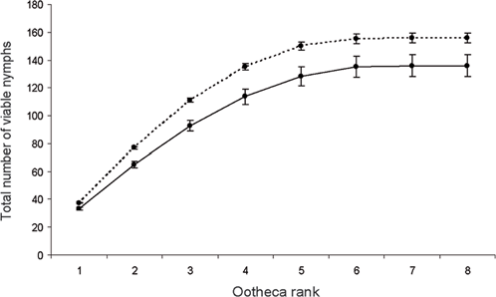
Reproductive success of inbred and outbred pairs. Cumulative mean number of viable nymphs produced per female (exp. 2a-c) in relation to ootheca production rank, until death of female. Comparisons of total numbers of offspring between inbred (63 replicates) and outbred pairs (212 replicates) were significant for each ootheca rank (Wilcoxon test, P<0.05). Solid line: inbred pairs; dotted line: outbred pairs. Means±SE are shown.

To estimate the reproductive success of pairs, successfully mated females (exp. 2a–c) were maintained isolated in their experimental boxes until their death. The total number of viable nymphs produced by females was significantly influenced by their lifespan and by their relatedness to males (ANCOVA, lifespan: F_1, 275_ = 38.02, P<0.01; relatedness: F_1, 275_ = 11.69, P<0.01). Inbred pairs produced less offspring than outbred pairs (Figure 4). Numbers of first ootheca offspring differed significantly between inbred and outbred females. This difference remained significant for the following oothecae and reached 13.54% at the females' death (Figure 4). The interaction between the two independent variables was not significant (lifespan×relatedness: F_1, 275_: 1.16, P = 0.28).

## Discussion

Our two complementary experiments (exp. 1–2) demonstrated that both male and female *B. germanica* exert some degree of mate choice. Although they use different mate selection criteria at different steps during the mating sequence, resulting pairings reveal selection for close inbreeding avoidance. Our reproductive success data support the adaptive value of this mating system.

### Mate choice by males

Males in large *B. germanica* aggregates have opportunities to choose among simultaneously calling females with regard to their relatedness. In our experiments, males clearly biased their courting investment towards non-siblings female lures, suggesting male mating preference based on their relatedness to females. As they persistently preferred to court non-sibling partners, even when they were male lures, we can discard the hypothesis of cryptic information transfer (either chemical or mechanical) from females to males that would influence male decision. Consequently, this validates the hypothesis of precopulatory mate choice by males and *B. germanica* can be added to the growing list of species for which male mate selectivity has been reported [15]–[20], [31], [50]–[52]. Contrary to many systems where males show a preference for a given female phenotype, a system based on relatedness, like the one we evidence here, includes no “best females”, as the quality of each female is primarily linked to her genetic relatedness to the encountered males.

Our results also yield information about the role of antennal contacts that occur at the beginning of the mating sequence. When males encountered female lures, a few antennal contacts were enough to trigger courting, whereas in the presence of male lures antennal contacts increased and courting decreased. These behavioural differences indicate that males assessed the sex of the potential partner through perception of the contact sex pheromone during the antennal contact phase. In addition, although our experiment did not allow us to examine possible roles of airborne pheromones, the fact that the first olfactometer arm visited was chosen randomly, even when the two lures differed in their relatedness, indicates that they did not select their mates via an airborne message. This result confirms our previous results in *B. germanica* [38] and is in agreement with reports on many other insect species [53]–[56] where kin recognition cues are carried by cuticular hydrocarbons and perceived through antennal contacts. At the beginning of the mating sequence, antennal contacts allow males to assess key information (sex and relatedness) concerning the quality of the encountered partner and this helps them to decide whether to invest or not in vigorous courting displays. Antennal contacts may thus constitute a phase of chemical assessment of female quality rather than courtship phase sensu stricto.

### Mate choice by females

After having attracted males in situ, calling *B. germanica* females are simultaneously courted by several potential partners and thus have the opportunity to exert actively a selection. In our experiments, females mated with the male that courted them the most vigorously. The absence of precopulatory intersexual agonistic interactions in *B. germanica* [32], [35] indicates that matings are not the result of potential male sexual coercion. Our data reveal a precopulatory female choice for males displaying the most vigorous courtships. As courtship vigour is strongly linked to male relatedness to females, females mated more frequently with non-sibling males than with sibling males. They thus seem to assess male quality by using, in part, courtship vigour as a phenotypic indicator. Nevertheless we cannot exclude that, as males, females use genetic relatedness assessment during antennal contacts with encountered partners to bias their choice towards non-siblings.

This preference for partners displaying the most intensive courtship signals has been observed in many other species where courtship vigour is used as a phenotypic indicator of fecundity [5]. Females that mated with siblings produced up to 13.54% less viable nymphs than females that mated with non-siblings. By choosing males displaying the most vigorous courtships, females enhance their probability to mate with non-siblings and thus to avoid reproductive success impairments. As *B. germanica* females mate only once [46], this stresses the importance of being selective.

### Conclusions

Our data revealed that *B. germanica* males and females express mating preferences. Both sexes base their mate choice on different criteria and select their partners at different steps of the mating sequence. After responding to the calling sex pheromone, males initiate antennal contacts with potential partners that give them the necessary information to assess their sex, their relatedness and to decide to invest or not in courtship. Males indicate their motivation to mate with less closely related females by displaying intense courtship sequences. As females primarily assess male quality through courtship intensity, they consequently choose the less closely related males. The resulting pairings are a consequence of mutual mate choice that favours close inbreeding avoidance. The impact on the reproductive success of pairs indicates the importance of inbreeding costs in this species and the adaptive value of this mating system. Our results suggest that this common inbreeding avoidance strategy of males and females could be a key for the preservation of genetic diversity in cockroach meta-populations.

For the first time, we present empirical evidence of mutual mate choice based on relatedness and leading to inbreeding avoidance. This suggests that *B. germanica* males and females do not fall into the sexual conflict zone initially predicted by Parker's inbreeding conflict model [26], [30] and recently revised by Kokko and Ots [29]. Cockroaches may thus fulfil the requirements for which it should pay both sexes to avoid inbreeding. Models predict that species that incur high inbreeding costs and/or that present symmetric reproductive investments by both sexes, should exert mutual selectivity. Although inbreeding costs have been estimated at 13.54% for *B. germanica,* our data did not allow us to quantify clearly other parameters linked to the reproductive investment of each sex (e.g. male courtship cost, spermatophore production cost, oothecae production cost) that are necessary to draw further conclusions. The emergence of mutual mate choice theory, coupled with the growing amount of empirical evidence, indicates the need to consider male selectivity as a confounding variable in studies of female mate choice. Our study stresses that detailed behavioural analyses of mating sequences are a good method to revisit mating systems. These investigations would be of primordial interest to test theoretical models and to improve the way we understand sex roles.
